# Single‐heartbeat cardiac cine imaging via jointly regularized nonrigid motion‐corrected reconstruction

**DOI:** 10.1002/nbm.4942

**Published:** 2023-05-13

**Authors:** Gastao Cruz, Kerstin Hammernik, Thomas Kuestner, Carlos Velasco, Alina Hua, Tevfik Fehmi Ismail, Daniel Rueckert, Rene Michael Botnar, Claudia Prieto

**Affiliations:** ^1^ School of Biomedical Engineering and Imaging Sciences King's College London London UK; ^2^ Department of Computing Imperial College London London UK; ^3^ Institute for Artificial Intelligence and Informatics in Medicine, Klinikum rechts der Isar Technical University of Munich Munich Germany; ^4^ Medical Image and Data Analysis, Department of Diagnostic and Interventional Radiology University Hospital Tübingen Tübingen Germany; ^5^ Escuela de Ingeniería Pontificia Universidad Católica de Chile Santiago Chile; ^6^ Millennium Institute for Intelligent Healthcare Engineering Santiago Chile

**Keywords:** cardiac MR, CINE, compressed sensing, motion correction, single‐heartbeat

## Abstract

The aim of the current study was to develop a novel approach for 2D breath‐hold cardiac cine imaging from a single heartbeat, by combining cardiac motion‐corrected reconstructions and nonrigidly aligned patch‐based regularization. Conventional cardiac cine imaging is obtained via motion‐resolved reconstructions of data acquired over multiple heartbeats. Here, we achieve single‐heartbeat cine imaging by incorporating nonrigid cardiac motion correction into the reconstruction of each cardiac phase, in conjunction with a motion‐aligned patch‐based regularization. The proposed Motion‐Corrected CINE (MC‐CINE) incorporates all acquired data into the reconstruction of each (motion‐corrected) cardiac phase, resulting in a better posed problem than motion‐resolved approaches. MC‐CINE was compared with iterative sensitivity encoding (itSENSE) and Extra‐Dimensional Golden Angle Radial Sparse Parallel (XD‐GRASP) in 14 healthy subjects in terms of image sharpness, reader scoring (range: 1–5) and reader ranking (range: 1–9) of image quality, and single‐slice left ventricular assessment. MC‐CINE was significantly superior to both itSENSE and XD‐GRASP using 20 heartbeats, two heartbeats, and one heartbeat. Iterative SENSE, XD‐GRASP, and MC‐CINE achieved a sharpness of 74%, 74%, and 82% using 20 heartbeats, and 53%, 66%, and 82% with one heartbeat, respectively. The corresponding results for reader scoring were 4.0, 4.7, and 4.9 with 20 heartbeats, and 1.1, 3.0, and 3.9 with one heartbeat. The corresponding results for reader ranking were 5.3, 7.3, and 8.6 with 20 heartbeats, and 1.0, 3.2, and 5.4 with one heartbeat. MC‐CINE using a single heartbeat presented nonsignificant differences in image quality to itSENSE with 20 heartbeats. MC‐CINE and XD‐GRASP at one heartbeat both presented a nonsignificant negative bias of less than 2% in ejection fraction relative to the reference itSENSE. It was concluded that the proposed MC‐CINE significantly improves image quality relative to itSENSE and XD‐GRASP, enabling 2D cine from a single heartbeat.

## Graphical abstract

Motion‐Corrected Cine (MC‐CINE) estimates cardiac motion from the acquired data itself and incorporates the motion into the cine reconstruction, leading to improved performance. Single‐heartbeat MC‐CINE achieved a similar performance to conventional iterative SENSE and XD‐GRASP reconstructions using 20 heartbeats.

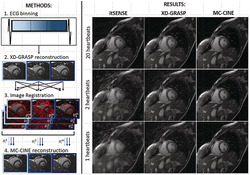

**Abbreviations**
ADMM: Alternating Direction Method of Multipliers
CMR: cardiovascular magnetic resonance
ECG: electrocardiogram
EDV: end diastolic volume
EF: ejection fraction
ESV: end systolic volume
GRICS: Generalized Reconstruction by Inversion of Coupled Systems
HD‐PROST: high‐dimensional undersampled patch‐based reconstruction
itSENSE: iterative sensitivity encoding
LV: left ventricle
MC‐CINE: Motion‐Corrected CINE
XD‐GRASP: Extra‐Dimensional Golden Angle Radial Sparse Parallel

## 1 INTRODUCTION

Cardiac cine imaging is a key application in cardiovascular magnetic resonance (CMR) and is considered the gold standard for cardiac function and morphology assessment.^1^ Cardiac cine imaging allows for the visualization of wall motion abnormalities, wall thickness, and measurement of quantitative metrics like ejection fraction (EF) that have diagnostic and prognostic importance.^2^ Cardiac cine imaging is commonly acquired in 2D, during breath‐hold to minimize artefacts from respiratory motion. Acquiring fully sampled images at the resolutions required in the clinic for each cardiac phase typically requires more than one heartbeat, therefore most methods rely on retrospective electrocardiogram (ECG) gating. Cardiac motion is assumed to be periodic and therefore different segments of k‐space may be acquired in separate heartbeats and grouped (binned/gated) to each corresponding phase, until all cardiac phases are sufficiently sampled (according to the reconstruction method employed). Over the last 20 years, considerable efforts have been made towards improving reconstruction performance, which may be leveraged into reduced scan time and/or improved resolution/coverage.

Many of these methods aim to exploit temporal redundancies in the cine data. k‐t SENSE^3^ and k‐t GRAPPA^4^ were two of the first approaches to combine these ideas with parallel imaging.^5^, ^6^, ^7^ Despite different formulations (*x‐f* space for k‐t SENSE, *k‐t* space for k‐t GRAPPA), both methods attempt to exploit correlations in k‐space and time simultaneously. Subsequent work has estimated a patient‐specific low‐dimensional temporal subspace via principal component analysis (PCA), k‐t PCA,^8^ providing a more efficient mechanism to exploit the redundant information in the data. Alternative approaches using partially separable functions to formulate subspace‐constrained reconstructions^9^ were also demonstrated to good effect. Following the dissemination of compressed sensing in MR,^10^ many approaches were proposed to further accelerate scan times, exploiting data redundancy as sparsity in some domain. The initial results of k‐t SPARSE^11^ demonstrated the sparse properties of cine in the Wavelet and temporal Fourier domains, and how these strategies could improve reconstruction performance. k‐t FOCUSS^12^ used a patch‐based motion estimation/compensation while imposing a solution with sparse residuals relative to a reference frame. Temporally regularized approaches featuring joint image/coil estimation and solved using nonlinear inverse reconstructions have also achieved very high acceleration factors.^13^ Approaches like MASTeR^14^ impose (Wavelet) sparsity on a set of motion‐compensated cardiac phases for improved sparsity in the temporal dimension. Patch‐based regularizers such as PRICE^15^ have been proposed, without requiring knowledge of motion or reference frames. Dictionary‐based approaches were also developed, aiming to learn transforms with improved sparsifying properties.^16^, ^17^, ^18^ Using similar ideas to,^8^, ^9^ a sparsity and low rank structure, k‐t SLR,^19^ presented further improvements by leveraging useful properties of the Karhunen Loève Transform. Low rank plus sparse decomposition approaches were considered in several MR applications,^20^, ^21^, ^22^ helping further separate background (low rank) and dynamic (sparse) components, leading to improved reconstruction performance. Ideas from MASTeR and low rank plus sparse models have also been combined,^23^ leveraging the L + S structure in motion‐corrected domains. Extra‐Dimensional Golden Angle Radial Sparse Parallel (XD‐GRASP)^24^ is a compressed sensing‐based approach that is comprised of a framework using self‐gating for binning into multiple motion states, followed by reconstruction of these data with spatial and temporal total variation regularization.

All of the methods described above focused on reconstructing motion‐resolved images without explicitly modeling cardiac motion into the forward model, although some incorporated motion as part of the regularization. Following the introduction of generalized motion‐corrected reconstructions for MR,^25^ motion‐corrected forward models have been employed to correct mainly for respiratory motion and enable free‐breathing cine imaging.^26^, ^27^, ^28^ In this work, we incorporated cardiac motion information into the reconstruction process to achieve breath‐hold cine from a single heartbeat. First, cardiac motion is incorporated into the forward model, such that the reconstruction of each cardiac phase includes data from the complete cardiac cycle. This leads to a substantial increase of k‐space information (~30 x) in the data consistency term, resulting in a better posed inverse problem. Second, cardiac motion is also used in the regularization term to increase the sparsification of the regularizer. The regularization term is a patch‐based denoiser, based on ideas from self‐similar patches^29^, ^30^ and motion‐aligned regularizers.^14^, ^31^ The proposed Motion‐Corrected CINE (MC‐CINE) enables an acceleration factor of ~20 x, allowing for cardiac cine imaging to be performed in a single heartbeat. The proposed approach was evaluated in 14 healthy subjects and compared with conventional iterative sensitivity encoding (itSENSE)^32^ and XD‐GRASP^24^ reconstructions.

## 2 METHODS

### 2.1 MC‐CINE framework

**FIGURE 1:**
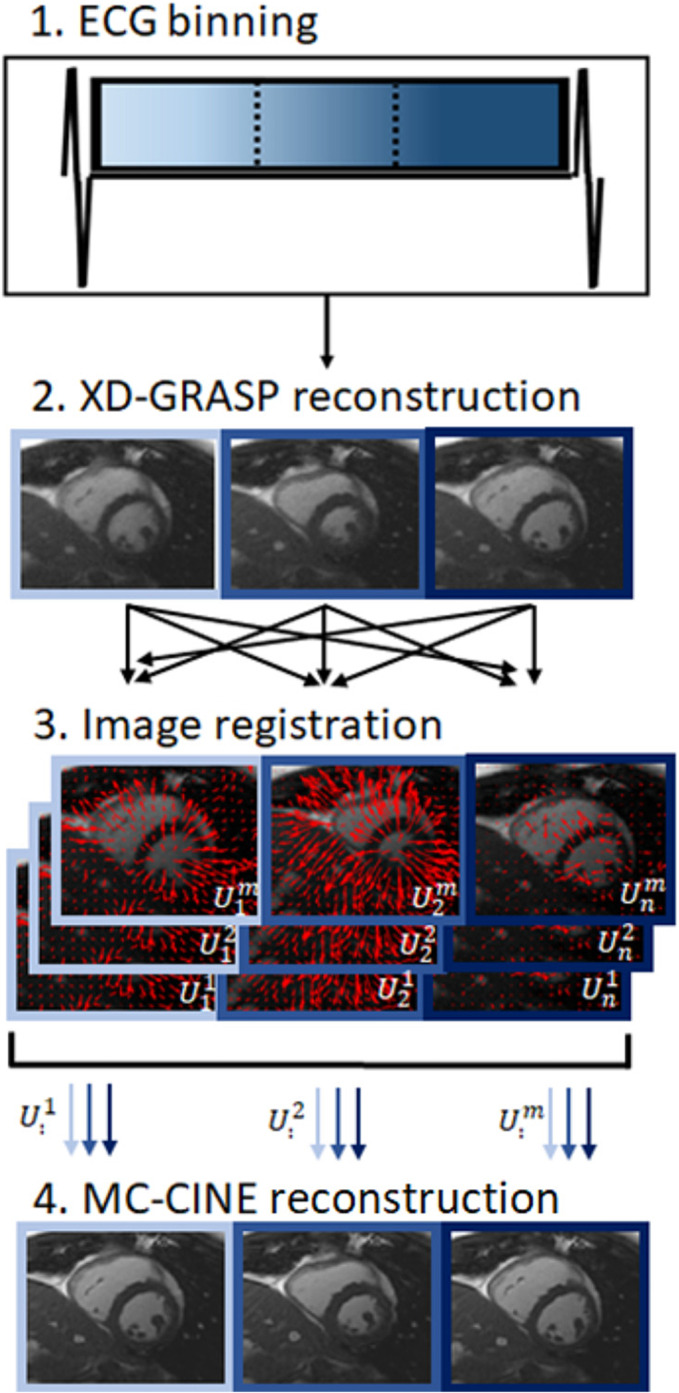
Diagram of the proposed MC‐CINE reconstruction framework. (1) Acquired data are binned into multiple cardiac phases, guided by ECG measurements, using a tiny golden radial trajectory. (2) An auxiliary cardiac‐resolved reconstruction is performed via XD‐GRASP. (3) Image registration is performed between each of the *m* cardiac phases to estimate *m* ^*2*^ motion fields. (4) Motion is incorporated into the forward model of the MC‐CINE, together with a nonrigidly aligned patch‐based regularizer, as described in Equation (1). Three representative phases are shown in the diagram; 30 phases were considered for the in vivo experiments. ECG, electrocardiogram; MC‐CINE, Motion‐Corrected CINE; XD‐GRASP, Extra‐Dimensional Golden Angle Radial Sparse Parallel.

**FIGURE 2:**
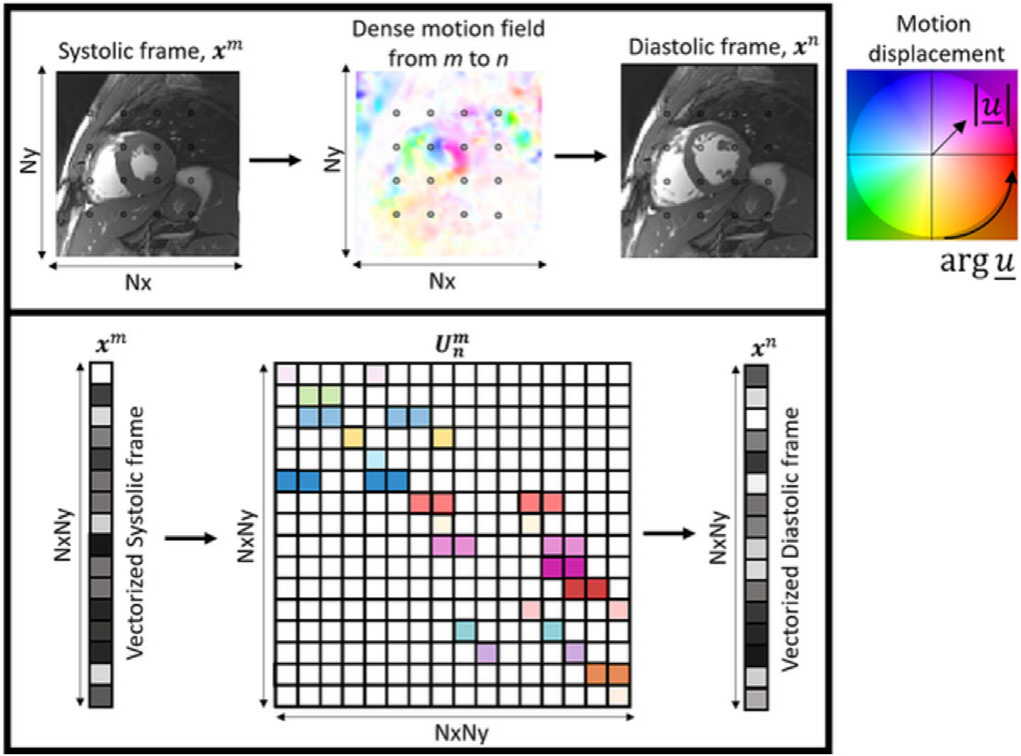
Pictorial diagram relating the motion representation between dense motion fields and sparse motion matrices. (Top) A systolic frame may be motion‐warped to a diastolic frame via a dense motion field. Each pixel is moved and interpolated accordingly to a Cartesian grid. Circles denote representative pixel locations used in the example for the sparse motion matrix below. (Bottom) Equivalent representation of both image frames and motion field, now cast as a sparse motion matrix. The process of motion warping is represented as a matrix–vector multiplication with a sparse matrix where the nonzero elements (colored) are the corresponding interpolation weights of the destination pixel(s).

The proposed reconstruction framework comprises four steps, as summarized in Figure 1: (1) ECG binning; (2) cardiac motion‐resolved reconstruction; (3) cardiac motion estimation; and (4) cardiac motion‐corrected reconstruction. Initially, data (acquired with a tiny golden radial trajectory) are retrospectively binned into equally long cardiac phases, as conventionally done. Although an ECG was employed in this study, alternative cardiac‐gating methods can also be used for binning. Thereafter, an auxiliary cardiac‐resolved reconstruction is performed, using XD‐GRASP.^24^ These intermediate images are used solely for nonrigid cardiac motion estimation using image registration,^33^, ^34^ where motion fields are estimated for every pair of motion states. Finally, the proposed MC‐CINE is performed, incorporating the estimated cardiac deformation fields into the encoding operator to reconstruct each cardiac phase. Considering a cine series with image size $N_{x}\times N_{y}$ and $N_{p}$ cardiac phases, and a corresponding k‐space with $N_{\mathrm{FE}}$, $N_{\mathrm{PE}}$, and $N_{c}$ dimensions along readout, phase encoding, and coil channel, this reconstruction is formulated as:

(1) $$\begin{array}{c} \hat{x}=\arg\;\min_{x}\;\frac{1}{2}{\left\|W\left(\text{AFCMx}-k\right)\right\|}_{2}^{2}+\lambda \sum_{b}{\left\|T_{b}\right\|}_{*},\\ s.t.\;T_{b}=Q_{b}\left(M^{H}I_{N_{p}}x\right) \end{array}$$

where $x={\left[x^{1},\ldots ,x^{m},\ldots ,x^{N_{p}}\right]}^{T}\in {\mathbb{C}}^{N_{x}N_{y}N_{p}\times 1}$ are the reconstructed CINE series where $x^{m}$ corresponds to the *m‐th* cardiac phase of the cine (for a total of $N_{p}$ cardiac phases), $\;W={\left[W^{1}\;\cdots \;W^{m}\;\cdots \;W^{N_{p}}\right]}^{T}\in {\mathbb{R}}^{N_{\mathrm{FE}}N_{\mathrm{PE}}N_{c}{N_{p}}^{2}\times N_{\mathrm{FE}}N_{\mathrm{PE}}N_{c}{N_{p}}^{2}}$ (where $W^{m}$ is a diagonal matrix) are soft‐weights for each cardiac phase *m*, $A=\left[\begin{array}{ccccc} A^{1} & \ldots & 0 & \cdots & 0\\ \vdots & \ddots & \vdots & \cdots & \vdots \\ 0 & \ldots & A^{m} & \cdots & 0\\ \vdots & \ldots & \ldots & \ddots & \vdots \\ 0 & \ldots & 0 & \ldots & A^{N_{p}} \end{array}\right]$ (where $A^{m}=\left[\begin{array}{ccccc} A_{1}^{m} & \ldots & 0 & \ldots & 0\\ \vdots & \ddots & \vdots & \ldots & \vdots \\ 0 & \ldots & A_{n}^{m} & \ldots & 0\\ \vdots & \ldots & \ldots & \ddots & \vdots \\ 0 & \ldots & 0 & \ldots & A_{N_{p}}^{m} \end{array}\right]$, and $A_{n}^{m}\in {\mathbb{R}}^{N_{\mathrm{FE}}N_{\mathrm{PE}}N_{c}{N_{p}}^{2}\times N_{\mathrm{FE}}N_{\mathrm{PE}}N_{c}{N_{p}}^{2}}$ is a diagonal matrix) corresponds to the k‐space sampling for each motion state *n* being used to reconstruct cardiac phase *m*, $F\in {\mathbb{C}}^{N_{\mathrm{FE}}N_{\mathrm{PE}}N_{c}{N_{p}}^{2}\times N_{x}N_{y}N_{c}{N_{p}}^{2}}$ is the Fourier transform, $C\in {\mathbb{C}}^{N_{x}N_{y}N_{c}{N_{p}}^{2}\times N_{x}N_{y}{N_{p}}^{2}}$ are the coil sensitivities, $M=\left[\begin{array}{ccccc} U^{1} & \ldots & 0 & \cdots & 0\\ \vdots & \ddots & \vdots & \cdots & \vdots \\ 0 & \ldots & U^{m} & \cdots & 0\\ \vdots & \ldots & \ldots & \ddots & \vdots \\ 0 & \ldots & 0 & \ldots & U^{N_{p}} \end{array}\right]$ (where $U^{m}={\left[\begin{array}{ccccc} U_{1}^{m} & \cdots & U_{n}^{m} & \cdots & U_{N_{p}}^{m} \end{array}\right]}^{T}$, and $U_{n}^{m}\in {\mathbb{R}}^{N_{x}N_{y}\times N_{x}N_{y}}$ is the motion field between phases *m* and *n*, cast as sparse matrices) contains all the forward motion fields from each cardiac phase *m* towards every cardiac phase *n*, $k={\left[k^{1},\ldots ,k^{m}\ldots ,k^{M}\right]}^{T}\in {\mathbb{C}}^{N_{\mathrm{FE}}N_{\mathrm{PE}}N_{c}{N_{p}}^{2}\times 1}$ are the acquired k‐space data, λ is the weight that balances the data consistency and regularization terms, $Q_{b}$ generates a 3D tensor $T_{b}$ (from the cardiac phase‐aligned series $M^{H}I_{m}x$) of voxels associated with the *b‐th* voxel, by concatenating local voxels (within a local patch) along the first dimension, nonlocal voxels (from patches that exhibit structural similarity with the patch around *b*) and (motion‐aligned) cardiac phases and $I_{N_{p}}\in {\mathbb{R}}^{N_{x}N_{y}{N_{p}}^{2}\times N_{x}N_{y}N_{p}}$ is a stack of identity matrices. ${\left\|\cdot \right\|}_{2}\;\text{and}\;{\left\|\cdot \right\|}_{*}$ refer to the L2 and nuclear norms, respectively. The soft‐weights W in the motion‐corrected reconstruction are valued between zero and one, for each k‐space line and each motion state, allowing any piece of data to belong partially to multiple motion states. Soft‐weighting has been previously used in MR reconstruction in the context of motion compensation, weighting data according to some motion signal to reduce aliasing artefacts at the expense of potential blurring.^35^, ^36^, ^37^ General motion fields are considered, which require a matrix size of [ $N_{x}$, $N_{y}$, 2] in 2D. Motion fields are expressed as sparse matrices $U_{n}^{m}$ of size [ $N_{x}N_{y}$, $N_{x}N_{y}$], where the nonzero values in each row correspond to the interpolation weights associated each displacement.^38^ The diagram in Figure 2 relates the dense motion field representation to the sparse matrix representation, which motion‐warps images using simple matrix–vector multiplications. The proposed forward model considers the reconstruction of each cardiac phase $x^{m}$ as a motion‐corrected reconstruction using all the acquired data (from various cardiac phases). The operator **M** in the encoding operator includes a set of $U^{m}$, each containing all the motion fields pertaining to the *m‐th* cardiac phase as a reference. Therefore, the same raw data get used *m* times to reconstruct *m* different cardiac phases, according to the underlying motion captured in each $U^{m}$. $M^{H}$ will approximate the inverse motion model, thus warping a set of cardiac phases towards a reference phase *m* (and will do so multiple times considering each cardiac phase as the reference). The regularization term, an extension of the previously proposed high‐dimensional undersampled patch‐based reconstruction (HD‐PROST),^39^ states that image patches of the reconstructed cine, once motion is warped to a common motion state, can be assembled into a local tensor with known low rank properties that are exploited to suppress noise and/or incoherent aliasing. At its core, the method relies on motion‐corrected reconstructions,^25^ which are generally ill‐posed (even in “fully sampled” acquisitions). In addition to parallel imaging, soft‐weighting^35^, ^37^ is incorporated into the forward model to help in that regard, effectively trading off residual aliasing with residual blurring. Further suppression of residual aliasing is afforded by the regularization. The problem in Equation (1) can be stated as an unconstrained optimization and solved via the Alternating Direction Method of Multipliers (ADMM).^40^

### 2.2 In vivo experiments

The proposed cardiac MC‐CINE approach was evaluated in 14 healthy subjects (age = 31 ± 3 years, five females) at 1.5 T (Ingenia, Philips, Best, The Netherlands) using a 28‐channel cardiac coil. The study was approved by the institutional review board and written informed consent was obtained from all subjects according to institutional guidelines. Imaging parameters included one short axis slice; field of view (FOV) = 256 x 256 mm^2^ (considering inherent 2 x frequency encoding oversampling of radial trajectories); 8 mm slice thickness; resolution = 2 x 2 mm^2^; TE/TR = 1.16/2.3 ms; b‐SSFP readout; radial tiny golden angle of ~23.6°^41^, ^42^, ^43^; flip angle 60°; 8960 radial spokes acquired; nominal scan time ~20 s; breath‐hold acquisition. Data were reconstructed to produce 30 cardiac phases using itSENSE, XD‐GRASP, and the proposed MC‐CINE with three acceleration factors (relative to the radial Nyquist fully sampled value of ~384 radial spokes) of R ~ 1.3 x (all acquired data, corresponding to 8960 spokes), R ~ 13.5 x (one 10th of the data, corresponding to the last 896 spokes acquired), and R ~ 26.9 x (one 20th of the data, corresponding to the last 448 spokes acquired), resulting in effective acquisition times of ~20, ~2, and ~1 s, respectively. The undersampled datasets used for R = 13.5 and R = 26.9 came from the same single ECG‐gated 20‐s breath‐hold long continuous radial acquisition with tiny golden angle ordering, to improve the validity of the comparisons and minimize incidental sources of artefacts (e.g., respiratory drift during breath‐holds).

### 2.3 Reconstruction parameters

Data were binned into 30 equally separated cardiac phases by scaling bin widths according to the cardiac cycle duration. Soft‐weights were linearly decaying with a range of 50% of each cardiac bin width (Figure S4). Coil maps were estimated from the acquired data via ESPIRiT.^44^ Iterative SENSE (in‐house implementation of^32^) used six iterations and was solved with the Conjugate Gradient algorithm. XD‐GRASP used 10 iterations, temporal total variation regularization $\lambda_{t}=2\times 10^{-2}\;\max\left|x\right|$, spatial total variation regularization $\lambda_{s}=2\times 10^{-3}\;\max\left|x\right|$, and was solved with the Nonlinear Conjugate Gradient algorithm, following the code provided by the corresponding study.^24^ Image registration was performed with NiftyReg,^34^ using normalized local cross correlation, a control point spacing of two pixels for the B‐spline free‐form deformation motion model, bending, and elasticity penalties of $1\times 10^{-4}$. Motion fields were cast as sparse matrices considering linear interpolation (i.e., up to four interpolation weights per pixel). MC‐CINE used a regularization strength of $5\times 10^{-8}\;\max\left|x\right|$, linear interpolation was considered for the motion fields, five‐pixel patches for PROST regularization, and the problem was solved with ADMM, using five inner (Conjugate Gradient) iterations and three outer ADMM iterations. All parameters were defined based on inspection of two representative cases at R = 1.3 (no outlier corrections were considered). Reconstructions were performed on a Linux workstation with 12 Intel Xeon X5675 (3.07 GHz) processors and 200 GB RAM. Code was implemented in MATLAB, with the exception of PROST, NiftyReg, and ESPIRiT (C++). Each method (itSENSE, XD‐GRASP, and MC‐CINE) was reconstructed with acceleration factors of R = 1.3, R = 13.5, and R = 26.9. Considering the “fully sampled” case (R = 1.3), itSENSE took ~5 min, XD‐GRASP took ~2 h 40 min, and MC‐CINE took ~26 h, with ~2 h 40 min for auxiliary motion‐resolved reconstructions, ~13 h 50 min for motion estimation, and ~9 h 30 min for motion‐corrected reconstruction.

### 2.4 Image analysis

Image edge sharpness between the myocardium and the blood pool was measured in a semiautomated fashion along the entire left ventricle (LV) (360°) for each cardiac phase. The blood/myocardium border was detected via a segmentation routine (which was verified and manually corrected when required); 360 radial lines were automatically drawn, and edge sharpness was measured in the border. Sharpness was measured similar to previous studies,^30^, ^45^ such that $\text{sharpness}={\left|p_{80}-p_{20}\right|}^{-1}\times 100\left(\%\right)$, where $p_{80}$ ( $p_{20}$) is the pixel location at 80% (20%) of the maximum intensity along the 1D line being evaluated.

Two readers, with 12 and two years of experience in cardiac MR, were asked to assess the image quality of itSENSE, XD‐GRASP, and MC‐CINE at R = 1.3, R = 13.5, and R = 26.9. First, they were asked to score image quality according to the following scale: 5 ‐ excellent image quality: no visible aliasing, blurring, or noise artefacts (no impact on diagnostic confidence); 4 ‐ good image quality: minor aliasing, blurring, or noise artefacts (minimal impact on diagnostic confidence); 3 ‐ OK image quality: some aliasing, blurring, or noise artefacts (minor loss of diagnostic confidence); 2 ‐ mediocre image quality: considerable aliasing, blurring, or noise artefacts (considerable loss of diagnostic confidence); 1 ‐ poor image quality: significant aliasing, blurring, or noise artefacts dominate (major loss of diagnostic confidence/nondiagnostic). Half‐marks were allowed. Additionally, readers were asked to rank each reconstruction from 1 (worst) to 9 (best).

Single‐slice end systolic volume (ESV), end diastolic volume (EDV), and derived ejection fraction (EF) were computed in a semiautomated fashion based on the single 2D slice acquired. Statistical significance for reader scores and rankings was assessed with the Wilcoxon signed‐rank test; the remaining metrics were assessed via a two‐sample Student's *t*‐test, considering p≤0.05 as significant, relative to the reference itSENSE and also between MC‐CINE and XD‐GRASP at corresponding accelerations. Data and code will be made available from the authors upon reasonable request.

## 3 RESULTS

### 3.1 In vivo experiments

**FIGURE 3:**
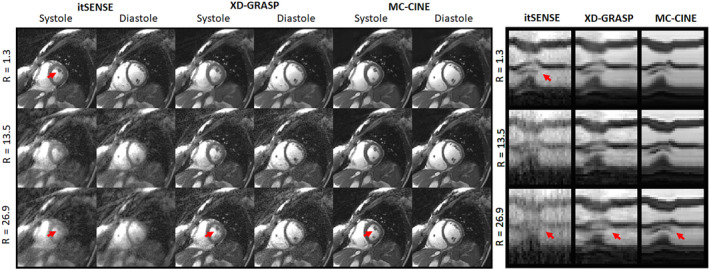
Systolic and diastolic cardiac CINE frames for representative subject A, reconstructed with itSENSE, XD‐GRASP, and the proposed MC‐CINE at three acceleration factors. Corresponding 1D temporal profiles are depicted in the right‐side panel. Blurring and incoherent aliasing are visible at higher accelerations for XD‐GRASP and especially itSENSE. MC‐CINE at R = 26.9 achieves similar image quality to reference itSENSE at R = 1.3 (arrows). itSENSE, iterative sensitivity encoding; MC‐CINE, Motion‐Corrected CINE; XD‐GRASP, Extra‐Dimensional Golden Angle Radial Sparse Parallel.

**FIGURE 4:**
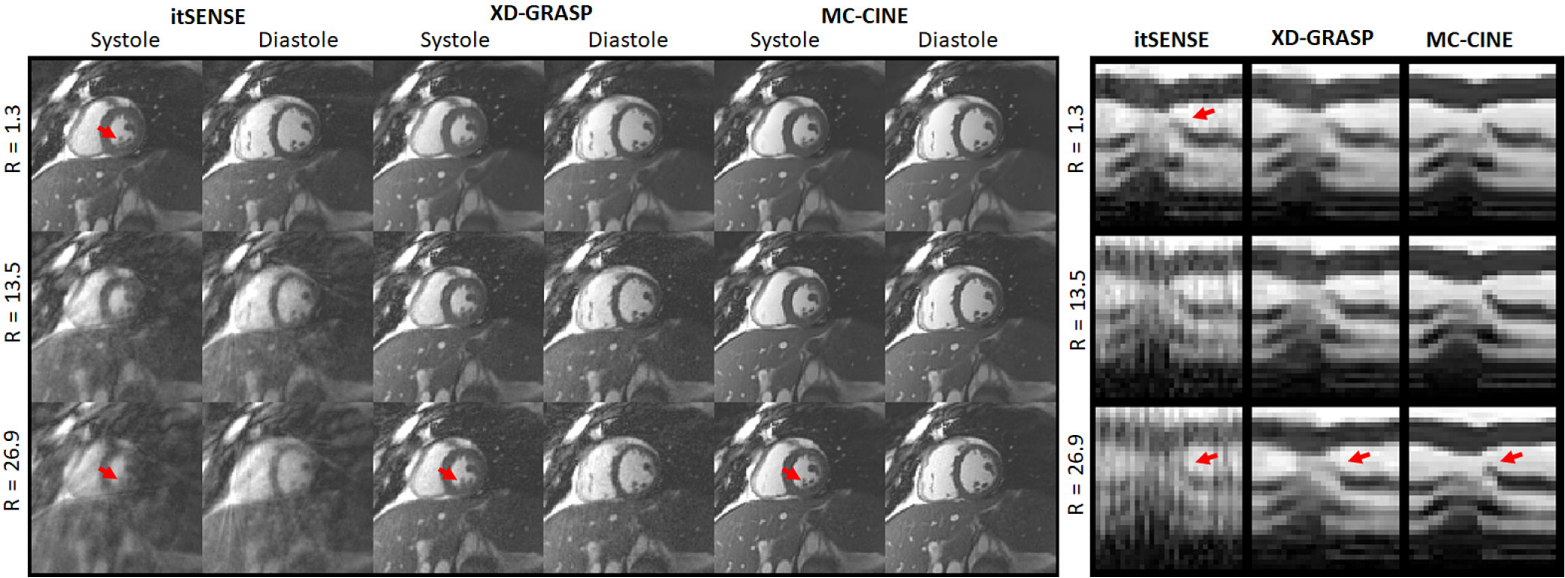
Systolic and diastolic cardiac CINE frames for representative subject B, reconstructed with itSENSE, XD‐GRASP, and the proposed MC‐CINE at three acceleration factors. Corresponding 1D temporal profiles are depicted in the right‐side panel. Similar to Figure 3, itSENSE image quality drops considerably at higher acceleration when only two heartbeats (R = 13.5) or one heartbeat (R = 26.9) is considered. XD‐GRASP recovers some image quality, but residual blurring and aliasing remain. The highest image quality (and temporal fidelity) is consistently achieved with MC‐CINE. itSENSE, iterative sensitivity encoding; MC‐CINE, Motion‐Corrected CINE; XD‐GRASP, Extra‐Dimensional Golden Angle Radial Sparse Parallel.

Cardiac cine frames for representative subjects A and B are shown in Figures 3 and 4 (respectively), reconstructed with itSENSE, XD‐GRASP, and the proposed MC‐CINE for R = 1.3, R = 13.5, and R = 26.9 (one heartbeat); moreover, corresponding 1D temporal profiles for each corresponding reconstruction are included in the right‐side panel. The three methods produce similar image quality for R = 1.3, however, itSENSE produces considerable aliasing artefacts for R = 13.5 and R = 26.9. XD‐GRASP suppresses many of these aliasing artefacts, however, residual aliasing and blurring remain, especially at R = 26.9. MC‐CINE further improves on XD‐GRASP, removing most residual artefacts at every acceleration factor, consistently achieving high image quality. As such, MC‐CINE at R = 26.9 achieves a similar image quality to itSENSE at R = 1.3. Similar trends are observed when inspecting the 1D temporal profiles: at higher accelerations, considerable artefacts are present for itSENSE, blurring is visible for XD‐GRASP, and high image quality is maintained for MC‐CINE. Higher blurring effects were generally observed around systole. Animated cardiac CINE images for representative subjects A and B are shown in the supporting information (Movie S1 and S2, respectively), where the dynamics of the full cardiac cycle may be seen for all methods and acceleration factors considered in this study. Cardiac motion is correctly resolved for all methods at all accelerations and performance is similar to previous results. MC‐CINE at R = 26.9 produces similar image quality to itSENSE at R = 1.3. Estimated motion fields between systole and diastole for three acceleration factors are shown in Figures S1 and S2 for representative subjects A and B, respectively. Under this representation, the direction of the motion is denoted by color, while the magnitude of the displacement is denoted by the intensity of the color. Left ventricular radial motion and right ventricular translational motion can be observed. Some motion is present in surrounding organs (e.g., liver), while other regions, like chest wall, have virtually no motion.

**FIGURE 5:**
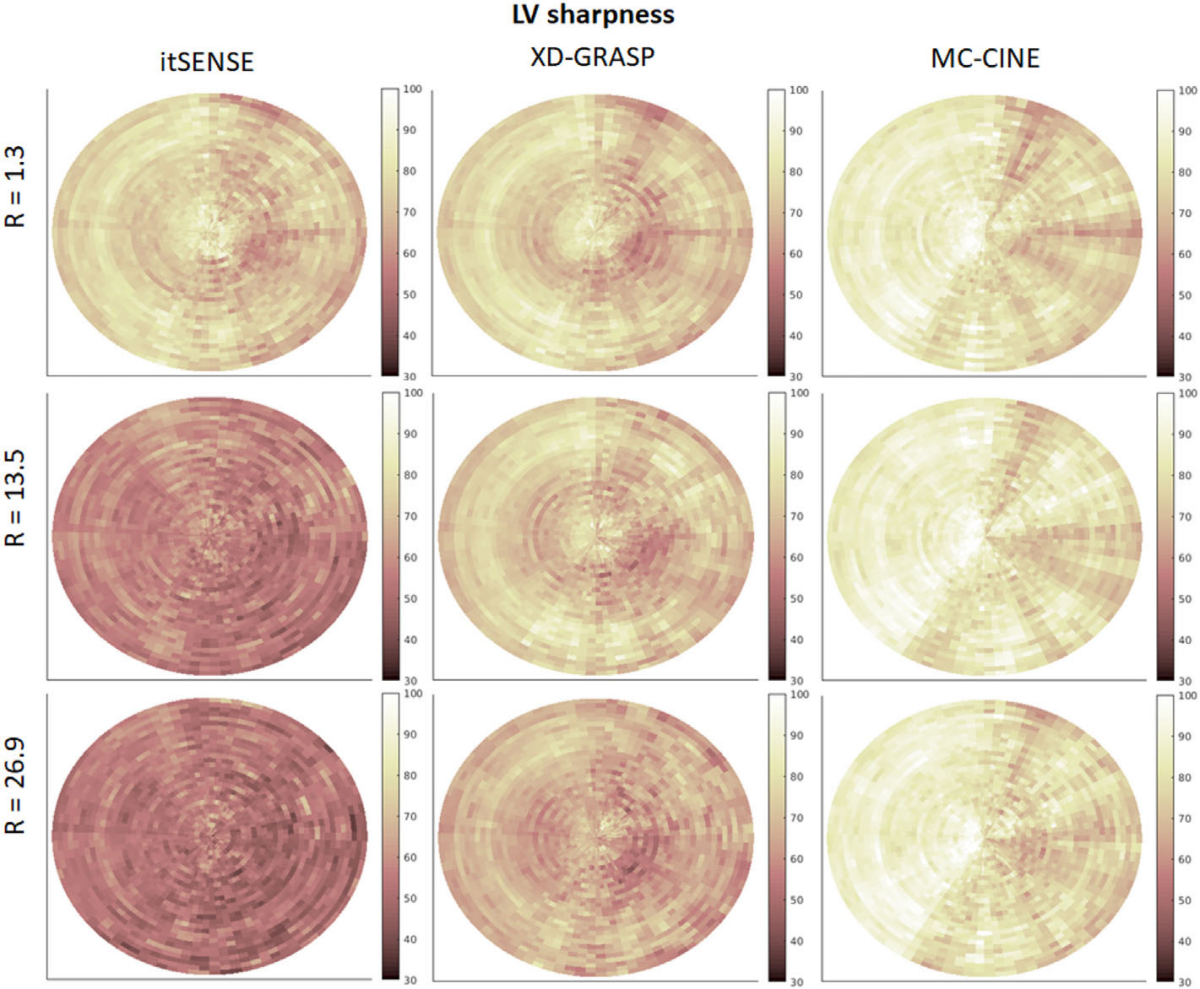
“Bullseye” style plot of the left ventricular endocardium sharpness for itSENSE, XD‐GRASP, and MC‐CINE at different acceleration factors, aggregated over the subject cohort. Each “ring” denotes the sharpness for a given cardiac phase, with the “end‐systolic” phase depicted in the innermost and the “end‐diastolic” phase depicted in the outermost. Minor and major losses of sharpness are observed for XD‐GRASP and itSENSE, respectively, whereas sharpness is maintained for MC‐CINE at every acceleration factor. itSENSE, iterative sensitivity encoding; MC‐CINE, Motion‐Corrected CINE; XD‐GRASP, Extra‐Dimensional Golden Angle Radial Sparse Parallel.

Image edge sharpness in the left ventricular blood/myocardium border is depicted in Figure 5, using a similar layout to the AHA “bullseye” plot.^46^ In this representation, each “ring” depicts a cardiac phase, with the innermost ring corresponding to the “end‐systolic” phase and the outermost ring corresponding to the “end‐diastolic” phase. As such, the angular coordinate corresponds to the angle of the line drawn from the center of the LV for sharpness measurements. In this representation, the left side corresponds to septal segments, the right side corresponds to lateral segments, the top side corresponds to anterior segments, and the bottom corresponds to inferior segments. Slightly inferior sharpness was generally observed in inferolateral segments and systolic phases. A considerable loss of sharpness was observed at higher accelerations for itSENSE. This trend was reduced with XD‐GRASP; however, some residual blurring remained, particularly in systolic (inner ring) phases and inferolateral segments. Finally, MC‐CINE maintained the same sharpness at higher accelerations in almost all the segments measured. The average LV sharpness (aggregated over the subject cohort) for itSENSE (R = 1.3, R = 13.5, and R = 26.9) was 74.6% ± 8.2%, 57.0% ± 6.3%, and 53.2% ± 6.4%, respectively; corresponding LV sharpness for XD‐GRASP was 74.2% ± 8.3%, 72.0% ± 8.2%, 65.8% ± 7.3%, respectively; corresponding LV sharpness for MC‐CINE was 82.5% ± 9.3%, 83.2% ± 9.5%, 82.1% ± 10.0%, respectively. XD‐GRASP produced significantly lower (*p* < 0.001) sharpness at R = 26.9 than itSENSE at R = 1.3; MC‐CINE produced significantly higher (*p* < 0.001) sharpness (at all acceleration factors) than itSENSE at R = 1.3. MC‐CINE achieved significantly higher sharpness (*p* < 0.001) than XD‐GRASP at every acceleration factor.

**FIGURE 6:**
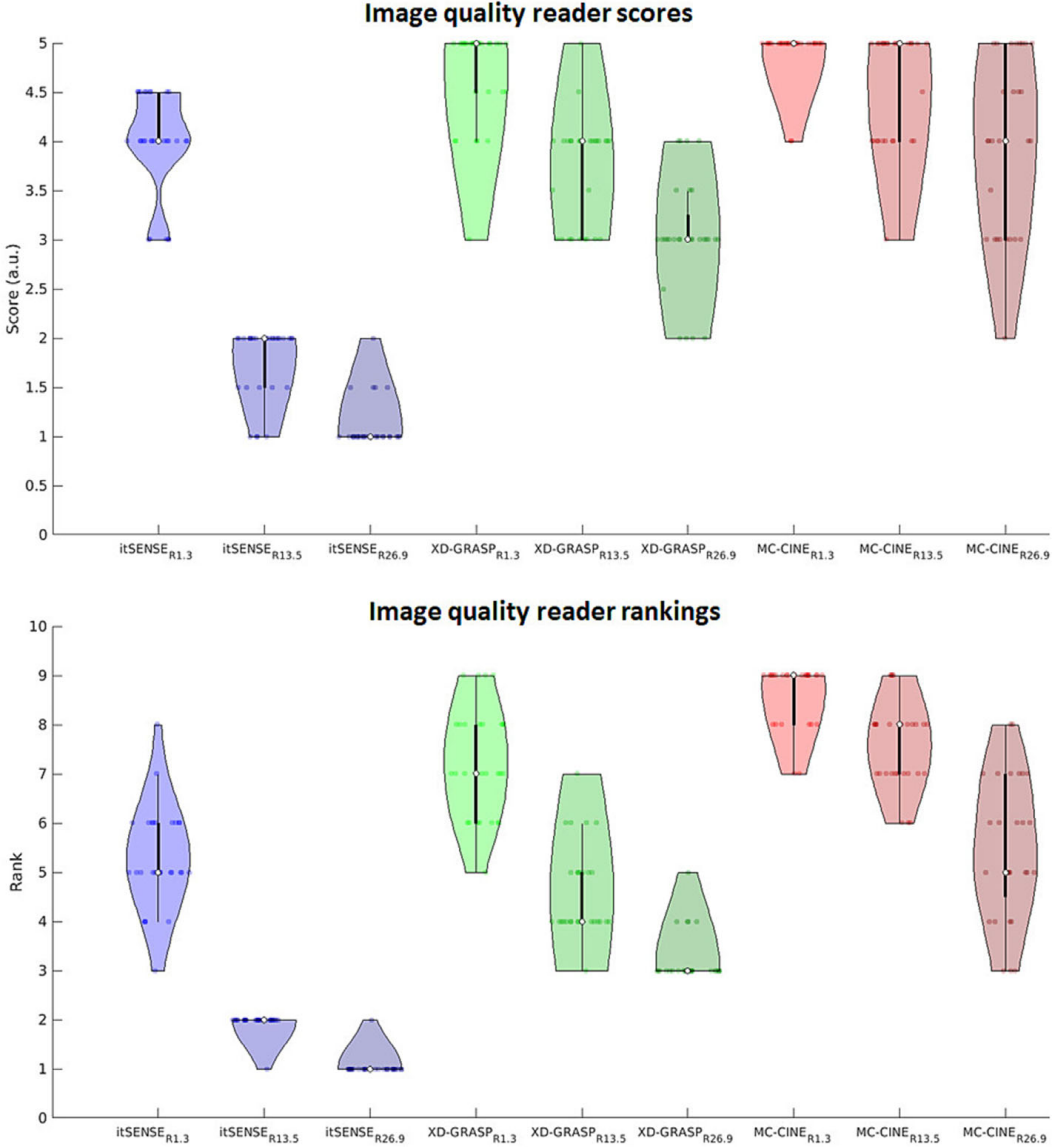
Violin plot of image quality scores and rankings by two readers for itSENSE, XD‐GRASP, and MC‐CINE at different accelerations. MC‐CINE consistently achieves higher image quality than XD‐GRASP at corresponding accelerations, whereas itSENSE admits major losses of image quality at higher accelerations. MC‐CINE is also ranked above XD‐GRASP for corresponding accelerations, with high‐acceleration itSENSE ranking at the bottom. In both metrics, MC‐CINE at R = 26.9 achieved similar performance to reference itSENSE at R = 1.3. itSENSE, iterative sensitivity encoding; MC‐CINE, Motion‐Corrected CINE; XD‐GRASP, Extra‐Dimensional Golden Angle Radial Sparse Parallel.

Reader scoring and ranking of image quality are presented in the violin plots of Figure 6 and are in general agreement with the other metrics assessed. Iterative SENSE suffered a large decrease in image quality scores and rankings at increased accelerations. XD‐GRASP was considerably more robust at higher accelerations, but still admitted losses in scores and rank. MC‐CINE presented higher robustness than XD‐GRASP with increasing accelerations, admitting small reductions in these metrics. MC‐CINE at R = 26.9 presented similar scores and rankings to itSENSE at R = 1.3. Averaged reader scores aggregated over the subject cohort for itSENSE (R = 1.3, R = 13.5, and R = 26.9) were (reported as median: [range]) [4: (3–5), 2: (1–2), 1.0: (1–2)], respectively; corresponding reader scores for XD‐GRASP were [5: (3–5), 4: (3–5), 3: (2–4)], respectively; corresponding reader scores for MC‐CINE were [5: (4–5), 5: (3–5), 4: (2–5)], respectively. Following the same notation for rankings, itSENSE rankings were [5: (3–8), 2: (1–2), 1: (1–2)]; XD‐GRASP rankings were [7: (5–9), 4: (3–7), 3: (3–5)]; and MC‐CINE rankings were [9: (7–9), 8: (6–9), 5: (3–8)]. XD‐GRASP produced significantly higher scores and rankings at R = 1.3 (*p* < 0.001), and significantly lower scores (and rankings) at R = 13.5 (*p* < 0.05) and R = 26.9 (*p* < 0.001) when compared with itSENSE at R = 1.3. MC‐CINE produced significantly higher scores (and rankings) at R = 1.3 (*p* < 0.001) and R = 13.5 (*p* < 0.001) than itSENSE at R = 1.3; no significant differences were observed between MC‐CINE at R = 26.9 and itSENSE at R = 1.3. MC‐CINE achieved significantly higher (*p* < 0.001) reader scores and rankings than XD‐GRASP at every acceleration factor.

**FIGURE 7:**
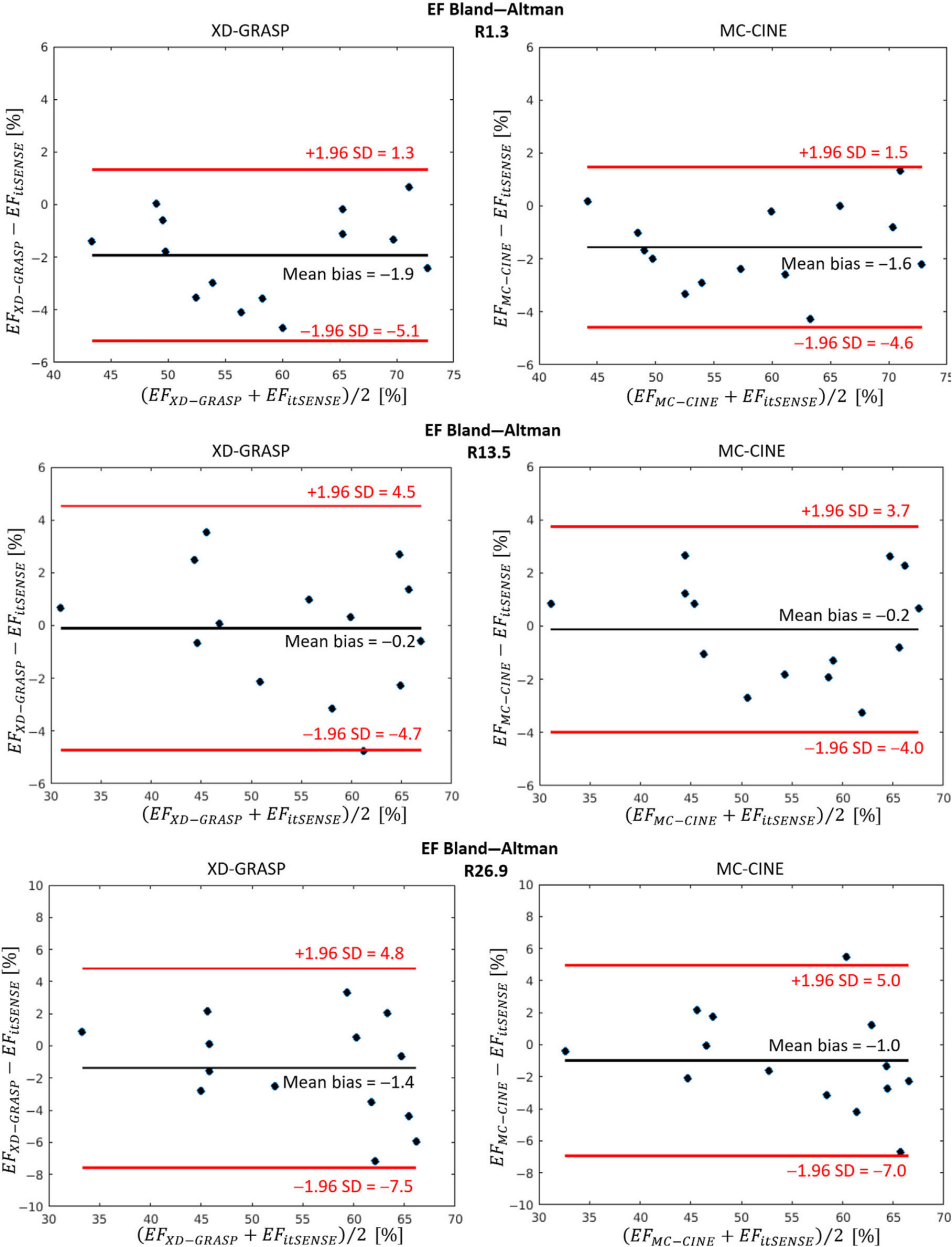
Bland–Altman plots of the ejection fraction (EF) for XD‐GRASP and MC‐CINE, relative to itSENSE, across all acceleration factors. XD‐GRASP and MC‐CINE both present small and comparable biases, resulting in a negative bias of < 2% for EF relative to the reference itSENSE with 20 heartbeats. Similar limits of agreement were also observed for both XD‐GRASP and MC‐CINE across all metrics. itSENSE, iterative sensitivity encoding; MC‐CINE, Motion‐Corrected CINE; XD‐GRASP, Extra‐Dimensional Golden Angle Radial Sparse Parallel.

Single‐slice ESV, EDV, and EF were measured for the three methods at each acceleration factor; a corresponding Bland–Altman plot for EF is shown in Figure 7 relative to itSENSE. Corresponding Bland–Altman plots for ESV and EDV can be found in Figures S2 and S3, respectively. At R = 1.3, a bias of +4.0 and +4.9 ml was observed in ESV for XD‐GRASP and MC‐CINE, respectively. Corresponding ESV biases for R = 13.5 were +0.4 and −0.7 ml, respectively. Corresponding ESV biases for R = 26.9 were +3.7 and +2.3 ml, respectively. A bias of −1.0 and +2.5 ml was observed in EDV for XD‐GRASP and MC‐CINE, respectively. Corresponding EDV biases for R = 13.5 were −0.2 and −1.9 ml, respectively. Corresponding EDV biases for R = 26.9 were +0.7 and −0.3 ml, respectively. Finally, biases of −1.9% and −1.6% were observed in EF for XD‐GRASP and MC‐CINE, respectively. Corresponding EF biases for R = 13.5 were −0.2% and −0.2%, respectively. Corresponding EF biases for R = 26.9 were −1.4% and −1.0%, respectively. Both XD‐GRASP and MC‐CINE produced similar variability in the measurements. No significant differences were observed in ESV, EDV, or EF for any method. The quantitative results for all metrics are compiled in Table 1.

**TABLE 1:** Summary of metrics evaluated to compare itSENSE, XD‐GRASP, and the proposed MC‐CINE. Single‐slice left ventricle (LV) to blood edge sharpness was computed in a semiautomated fashion; scores and rank were assessed by two readers; end systolic volume (ESV), end diastolic volume (EDV), and ejection fraction (EF) were computed in a semiautomated fashion.

	Sharpness (%)	Scores (a.u.)	Rank (a.u.)	LV ESV (ml)	LV EDV (ml)	LV EF (%)
itSENSE_R1.3_	74.6 ± 8.2	4.0 ± 0.5	5.3 ± 1.0	93.0 ± 38.0	228.1 ± 36.6	55.4 ± 9.2
XD‐GRASP_R1.3_	74.2 ± 8.3	4.7 ± 0.5^a^	7.3 ± 1.1^a^	96.8 ± 38.6	227.2 ± 36.7	53.5 ± 9.4
MC‐CINE_R1.3_	82.5 ± 9.3^a^ ^,^ ^c^	4.9 ± 0.3^a^ ^,^ ^c^	8.6 ± 0.6^a^ ^,^ ^c^	97.7 ± 39.7	230.5 ± 37.4	53.9 ± 9.5
itSENSE_R13.5_	57.0 ± 6.3^b^	1.8 ± 0.4^b^	1.9 ± 0.2^b^	107.3 ± 34.1	234.1 ± 36.4	54.4 ± 11.6
XD‐GRASP_R13.5_	72.0 ± 8.2	3.7 ± 0.6^b^	4.6 ± 0.9^b^	107.7 ± 32.6	234.4 ± 35.2	54.3 ± 10.4
MC‐CINE_R13.5_	83.2 ± 9.5^a^ ^,^ ^c^	4.6 ± 0.7^a^ ^,^ ^c^	7.5 ± 0.8^a^ ^,^ ^c^	106.5 ± 32.2	232.2 ± 34.3	54.3 ± 10.8
itSENSE_R26.9_	53.2 ± 6.4^b^	1.1 ± 0.3^b^	1.0 ± 0.2^b^	102.4 ± 30.3	231.6 ± 37.6	55.7 ± 10.9
XD‐GRASP_R26.9_	65.8 ± 7.3^b^	3.0 ± 0.6^b^	3.2 ± 0.5^b^	106.1 ± 30.0	232.2 ± 36.2	54.4 ± 9.8
MC‐CINE_R26.9_	82.1 ± 10.0^a^ ^,^ ^c^	3.9 ± 0.8^c^	5.4 ± 1.4^c^	104.6 ± 29.0	231.3 ± 35.5	54.8 ± 10.0

Abbreviations: itSENSE, iterative sensitivity encoding; MC‐CINE, Motion‐Corrected CINE; XD‐GRASP, Extra‐Dimensional Golden Angle Radial Sparse Parallel.

^a^ Denotes significantly superior.

^b^ Denotes significantly inferior (relative to the reference itSENSE at R = 1.3).

^c^ Denotes a significant difference between XD‐GRASP and MC‐CINE at each corresponding acceleration factor.

## 4 DISCUSSION

In this study we have evaluated the feasibility of a novel jointly regularized, cardiac motion‐corrected reconstruction for highly accelerated cardiac cine imaging enabling single‐heartbeat cine MRI. The proposed MC‐CINE reconstructs each cardiac phase via a motion‐corrected reconstruction,^25^ efficiently incorporating data from all phases into the reconstruction of each cardiac phase. Motion is estimated via auxiliary XD‐GRASP reconstructions, although other motion‐resolved methods can be considered instead. The reconstruction is further regularized by a patch‐based tensor denoiser^39^ applied to the cine series projected to a reference cardiac phase. Motion estimation and correction reconstruction frameworks have been under investigation for a long time, initially studied in knee and hand for real‐time MR.^47^ In previous studies, motion correction has been incorporated into cardiac cine reconstruction to correct respiratory motion and enable free‐breathing acquisitions.^26^, ^27^ Joint respiratory and cardiac motion correction for CINE was also proposed^48^ before using Generalized Reconstruction by Inversion of Coupled Systems (GRICS).^49^ The GRICS framework estimates motion as part of a joint motion‐estimation/motion‐correction reconstruction process, where motion is estimated based on the residual of the reconstruction and required at the time cardiac motion‐correction windows of ~200 ms. By contrast, MC‐CINE performs motion estimation/motion correction sequentially, estimates motion via image registration from auxiliary reconstructions, and incorporates data from the full cardiac cycle. Accurate motion estimation is key for quality MC‐CINE reconstructions, because errors in motion estimation can propagate into the final reconstructed images. Error propagation using similar frameworks has been preliminarily evaluated in other studies, indicating some robustness to errors (up to 20%) in estimated motion.^50^, ^51^, ^52^ Because GRICS estimates motion from the residual in the reconstruction, it may not be well disposed to highly undersampled problems such as single‐heartbeat CINE. Furthermore, GRICS and MC‐CINE approaches differ in regularization strategies. Similar regularization approaches to MC‐CINE have been considered in the past, leveraging information between similar patches along the cardiac cycle^15^, ^53^ or using motion to increase sparsity/low rankness,^23^, ^54^ for example. The regularization strategy employed here is similar to the recently proposed Multi‐Bin PROST,^30^ however, redundant patch‐based information is exploited in a motion‐aligned series and low‐rankness exploited in a 3D tensor, as opposed to exploiting low rankness of a 2D matrix assembled from a nonaligned motion series. More importantly, Multi‐Bin PROST does not incorporate motion information in the forward model, that is, it uses a motion‐resolved encoding operator instead.

The proposed MC‐CINE was compared with conventional XD‐GRASP and itSENSE at acceleration factors of R = 1.3, R = 13.5, and R = 26.9, corresponding to scans of approximately 20 heartbeats, two heartbeats, and one heartbeat, respectively. At high accelerations, itSENSE presented aliasing artefacts, as expected from radial undersampling. XD‐GRASP presented blurring and residual aliasing, as the temporal total variation regularization began to fail because of high undersampling factors. Blurring artefacts were particularly prominent around systolic phases, where the level of temporal sparsity is reduced. Additional suppression of noise and/or incoherent artefacts can be achieved with stronger regularization parameters, however, often at the expense of additional blurring (and potentially staircasing artefacts for total variation). MC‐CINE presented only minor residual incoherent artefacts at R = 26.9, arising from undersampling related aliasing, noise amplification, and residual errors in motion estimation. Regularization parameters (for both XD‐GRASP and MC‐CINE) were optimized for the case of R = 1.3 and fixed for all accelerations; slightly improved performance may be possible with tailored parameter selection. Further improvements on the MC‐CINE performance may be achieved by using a superior motion‐resolved reconstruction (e.g., deep learning‐based^55^, ^56^ instead of XD‐GRASP) for improved motion estimation and/or iterate between motion estimation and motion correction.^48^, ^49^, ^57^, ^58^

The performance of itSENSE, XD‐GRASP, and MC‐CINE was evaluated via image edge sharpness, reader scoring, and reader ranking, which were all in agreement. MC‐CINE consistently achieved the highest metrics at each acceleration level, significantly higher than XD‐GRASP. Moreover, MC‐CINE at R = 26.9 was not significantly different from itSENSE at R = 1.3 (whereas XD‐GRASP at R = 26.9 was significantly inferior). A general loss of sharpness, particularly in inferolateral segments during systole, was observed for XD‐GRASP and especially for itSENSE with increasing acceleration. Systolic phases are more likely to incur residual cardiac motion and potential temporal blurring from total variation regularization, and inferolateral segments are more susceptible to noise amplification at increased acceleration factors. Although some loss of sharpness was also expected for MC‐CINE at high accelerations, this was not observed. This is possibly attributable to a reduction of residual respiratory motion, that is, respiratory drift during breath‐holds, in R = 13.5 and R = 26.9, which partially compensates for any minor blurring introduced from residual aliasing and/or motion estimation errors. Additionally, MC‐CINE at R = 26.9 (one heartbeat) does not bin data across different heartbeats and therefore simultaneously corrects for cardiac and residual respiratory motion therein.

Small (nonsignificant) biases relative to itSENSE were observed for ESV, EDV, and EF with XD‐GRASP and MC‐CINE. The largest bias observed was of +4.0 and +4.9 ml for ESV with XD‐GRASP and MC‐CINE, respectively. This was probably because of the challenges in capturing the “end‐systolic” phase, as XD‐GRASP may produce some blurring from regularization that relies on neighboring phases. MC‐CINE uses these images for motion estimation and therefore may incur some bias in the motion estimation in addition to any errors that may arise during the image registration process. In this framework, motion is estimated via intensity‐based image registration, which may be sensitive to image artefacts. While incoherent artefacts, noise, and blurring generally have a small impact on the registration process, coherent aliasing or other sources of artefactual image contrast can propagate into motion estimation errors. Although no through‐plane motion was observed in the midventricular slices acquired, through‐plane motion could contribute to data inconsistency, in which case a 3D framework would have to be considered. Regardless, a negative bias of less than 2% EF was observed for both methods, which is in general agreement with recent compressed sensing and deep learning‐based CINE studies.^30^, ^56^

**FIGURE 8:**
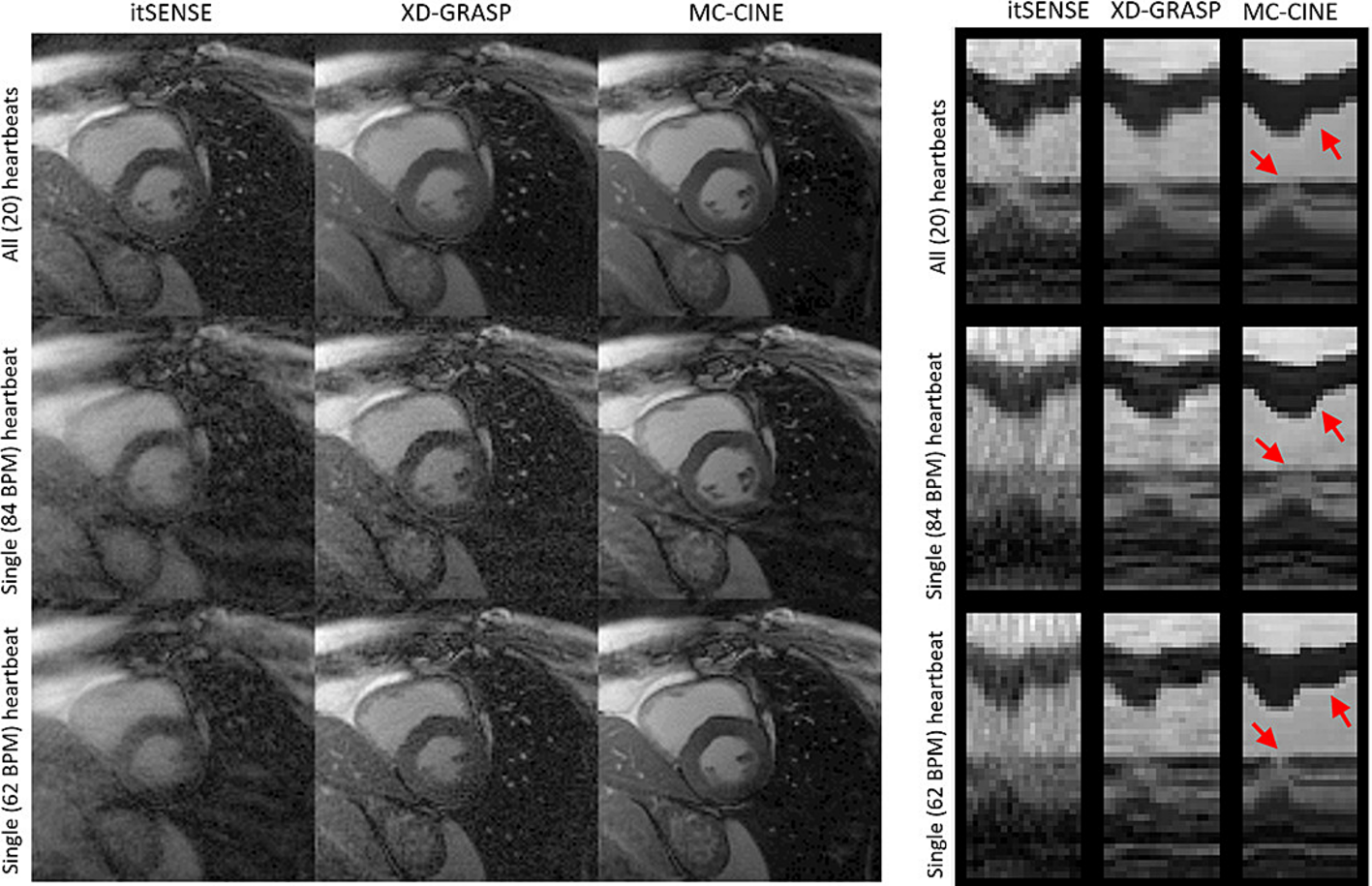
A systolic cardiac phase for subject C, reconstructed with itSENSE, XD‐GRASP, and MC‐CINE, using all the acquired data (20 heartbeats), only the fastest heartbeat (at 84 BPM), and only the slowest heartbeat (at 62 BPM). Corresponding 1D temporal profiles are depicted in the right‐side panel. We can see that the same systolic phase corresponds to different cardiac motion states depending on the heartbeat (or a blurring average in the case of 20 heartbeats), showing that there can be considerable differences in cardiac motion dynamics between different heartbeats (red arrows). Iterative SENSE and XD‐GRASP present blurring and aliasing artefacts due to high accelerations, whereas single‐heartbeat MC‐CINE accurately captures all the dynamic information. BPM, beats per minute; itSENSE, iterative sensitivity encoding; MC‐CINE, Motion‐Corrected CINE; XD‐GRASP, Extra‐Dimensional Golden Angle Radial Sparse Parallel.

**FIGURE 9:**
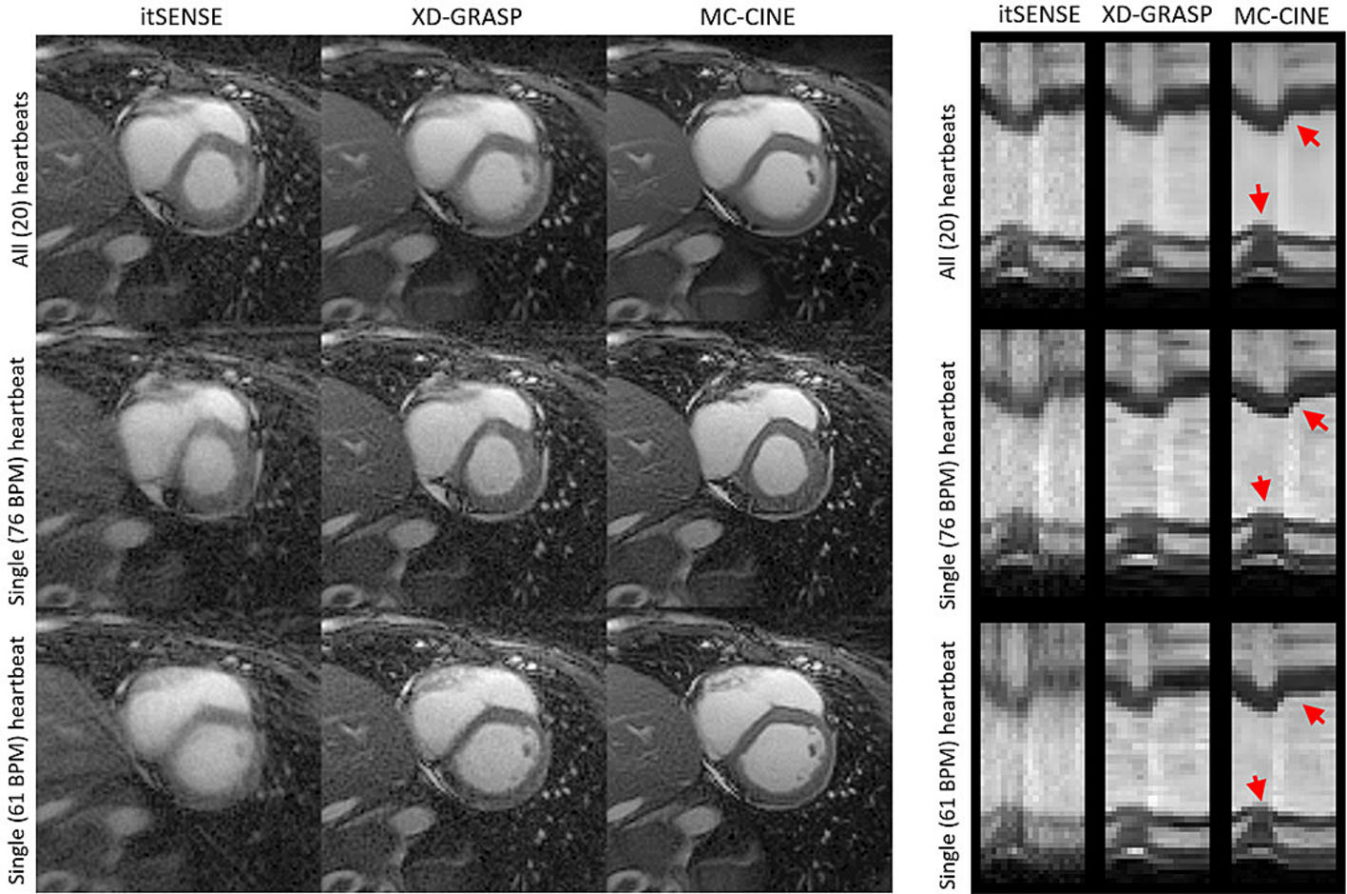
A systolic cardiac phase for subject D, reconstructed with itSENSE, XD‐GRASP, and MC‐CINE, using all the acquired data (20 heartbeats), only the fastest heartbeat (at 76 BPM), and only the slowest heartbeat (at 61 BPM). Corresponding 1D temporal profiles are depicted in the right‐side panel. Similar to Figure 8, residual cardiac blurring is present in all the reconstructions that use 20 heartbeats. When inspecting an end‐systolic frame reconstructed with only the fastest and the slowest heartbeat, it is clear that distinct motion states were binned into the same cardiac phase, leading to motion‐blurring artefacts. MC‐CINE achieves high quality cine from a single heartbeat, whereas XD‐GRASP and especially itSENSE suffer from blurring and aliasing artefacts. BPM, beats per minute; itSENSE, iterative sensitivity encoding; MC‐CINE, Motion‐Corrected CINE; XD‐GRASP, Extra‐Dimensional Golden Angle Radial Sparse Parallel.

Experimental results indicate that the proposed MC‐CINE at R = 26.9 (i.e., approximately a single heartbeat's worth of data) produces a similar cine series to itSENSE at R = 1.3 (i.e., 20 heartbeats). We believe that performing cine in a single heartbeat can be advantageous for several reasons. Very short breath‐holds are required (~1 s), which should be attainable for virtually any patient. Consequently, the method is insensitive to residual motion artefacts originating in respiratory drift that commonly occurs during breath‐holds. Single‐heartbeat MC‐CINE may be acquired without ECG triggering, because the ~1 s worth of data can be temporally resolved. Consequently, the reduced scan time improves patient throughput and comfort. Perhaps the most important property of single‐heartbeat MC‐CINE is that it does not assume periodicity of cardiac motion, making it more robust to heart rate variations that could otherwise produce cardiac motion artefacts when data are acquired over multiple cardiac cycles. We hypothesize that single‐heartbeat MC‐CINE could better resolve varying cardiac dynamics between different heartbeats (due to, e.g., arrhythmia or exercise MRI), which would otherwise be averaged into a multiple heartbeat acquired cine. Recent studies have investigated real‐time cine for patients with arrythmia, revealing an increase in image quality (because of reduction of cardiac motion artefacts) relative to conventional cine.^59^, ^60^ We have preliminarily investigated this possibility here by reconstructing a 20‐phase cardiac CINE of the fastest and the slowest heartbeats, with itSENSE, XD‐GRASP, and MC‐CINE. A representative cardiac phase reconstructed with all heartbeats, only the fastest heartbeat, and only the slowest heartbeat, is presented in Figures 8 and 9 for subjects D and E, respectively, along with corresponding 1D temporal profiles. Blurring artefacts are visible when all heartbeats are considered for reconstruction because the same cardiac phase can correspond to distinct motion states in different (e.g., fastest or slowest) heartbeats (particularly visible in Figure 9). MC‐CINE can be used to resolve each heartbeat independently, thereby resolving the unique dynamics of each cardiac cycle. Different cardiac dynamics for the fastest and slowest heartbeats can be observed in the corresponding 1D temporal profiles, as well as blurring artefacts in the case of all heartbeats (red arrows).

Our study has several limitations. The cohort evaluated was composed of young, healthy, and cooperative subjects, who tend to produce higher quality data than patients acquired in a clinical setting. The proposed approach was evaluated in midventricular, short axis slices; however, studies in additional orientations (e.g., two‐chamber, four‐chamber) would help characterize the performance of the proposed approach. Future work will validate MC‐CINE in other locations and orientations. The feasibility of the proposed approach was compared with a radial itSENSE using the same data (to minimize incidental artefacts); however, future work should also consider a Cartesian reference. The computational time of the proposed MC‐CINE is significantly longer than itSENSE or XD‐GRASP and requires substantial improvements for practical usage. The most time‐consuming steps were motion‐estimation and motion‐compensated reconstructions; however, both the tasks have been recently solved via efficient deep‐learning solutions,^61^, ^62^ which could be leveraged to substantially reduce the computational burden of MC‐CINE. ESV, EDV, and EF at R = 13.5 and R = 26.9 were in general agreement with measurements made at R = 1.3; however, we note that these measurements are more prone to errors caused by residual aliasing, especially for the reference itSENSE. This study was performed retrospectively, where accelerated reconstructions considered the last heartbeat(s) of the acquired data. Employing single‐heartbeat MC‐CINE in a prospective fashion will have to consider the initial transient state of the magnetization, which can be accomplished via dummy pulses to reach a steady state or by incorporating the magnetization dynamics directly into the reconstruction process. Because the proposed framework does not bin data from different heartbeats, single‐heartbeat MC‐CINE could be performed without ECG triggering or any other type of cardiac monitoring. Even if a multiple heartbeat sequence is considered, ECG‐less MC‐CINE is applicable because the cardiac phases are effectively temporally resolved, and this could be investigated in future work. The potential to perform CINE in a single heartbeat also enables new protocol options, such as increased resolution/coverage (e.g., whole‐heart coverage via multiple 2D slices in a single breath‐hold), or applications that require temporally resolved data (e.g., interventional MR). This study was performed using breath‐hold acquisitions; however, the proposed framework could also be used to correct for respiratory motion. Thus, free‐breathing, ECG‐less MC‐CINE could be explored in the future.

## 5 CONCLUSION

A novel reconstruction, MC‐CINE, is proposed, enabling single‐heartbeat cardiac cine imaging, by combining motion‐corrected reconstructions and nonrigidly aligned patch‐based regularization. Single‐heartbeat MC‐CINE achieved similar quality to itSENSE from 20 heartbeats, and outperformed XD‐GRASP at corresponding acceleration factors. Single‐heartbeat MC‐CINE temporally resolves data without grouping data between different heartbeats, which may help in capturing cardiac dynamics.

## Supporting information


**Figure S1.** MC‐CINE estimated motion fields between systole and diastole at three different acceleration factors, for representative subjects A and E. Contractile radial motion of the left ventricle and papillary muscles is apparent in the coloured representation of the motion. Motion of the right ventricle, liver and other surrounding organs can also be observed, in contrast to the chest wall which is almost absent of motion.

**Figure S2.** Bland–Altman plots of the End Systolic Volume (ESV) for XD‐GRASP and MC‐CINE, relative to iterative SENSE at multiple acceleration factors. XD‐GRASP and MC‐CINE both present small and comparable biases in ESV. Similar limits of agreement were also observed for both methods across all metrics.

**Figure S3.** Bland–Altman plots of the End Diastolic Volume (EDV) for XD‐GRASP and MC‐CINE, relative to iterative SENSE. XD‐GRASP and MC‐CINE both present small and comparable biases in EDV. Similar limits of agreement were also observed for both methods across all metrics.

**Figure S4.** Soft‐weight values used within the proposed approach, valued between zero and one. Data away from the centre of the phase is linearly weighted, allowing each data point to belong to multiple cardiac phases.

**Movie S1.** Animated CINE for representative subject A, reconstructed with iterative SENSE (column 1), XD‐GRASP (column 2) and the proposed MC‐CINE (column 3) at R = 1.3 (row 1), R = 13.5 (row 2) and R = 26.9 (row 3). The dynamics of the cardiac contraction are captured in every case, however residual streaking and noise amplification are visible for XD‐GRASP and especially iterative SENSE at higher accelerations. Single‐heartbeat MC‐CINE (at R = 26.9) achieves similar image quality to reference iterative SENSE at R = 1.3.

**Movie S2.** Animated CINE for representative subject E, reconstructed with iterative SENSE (column 1), XD‐GRASP (column 2) and the proposed MC‐CINE (column 3) at R = 1.3 (row 1), R = 13.5 (row 2) and R = 26.9 (row 3). Similar to SI Movie S2, MC‐CINE outperforms XD‐GRASP and iterative SENSE at each corresponding acceleration.
