# Supplementary figures and images for: Single‐heartbeat cardiac cine imaging via jointly regularized nonrigid motion‐corrected reconstruction
Source: NMR Biomed. 2023 May 13;36(9):e4942. doi: 10.1002/nbm.4942 (PMC10909414; doi:10.1002/nbm.4942)

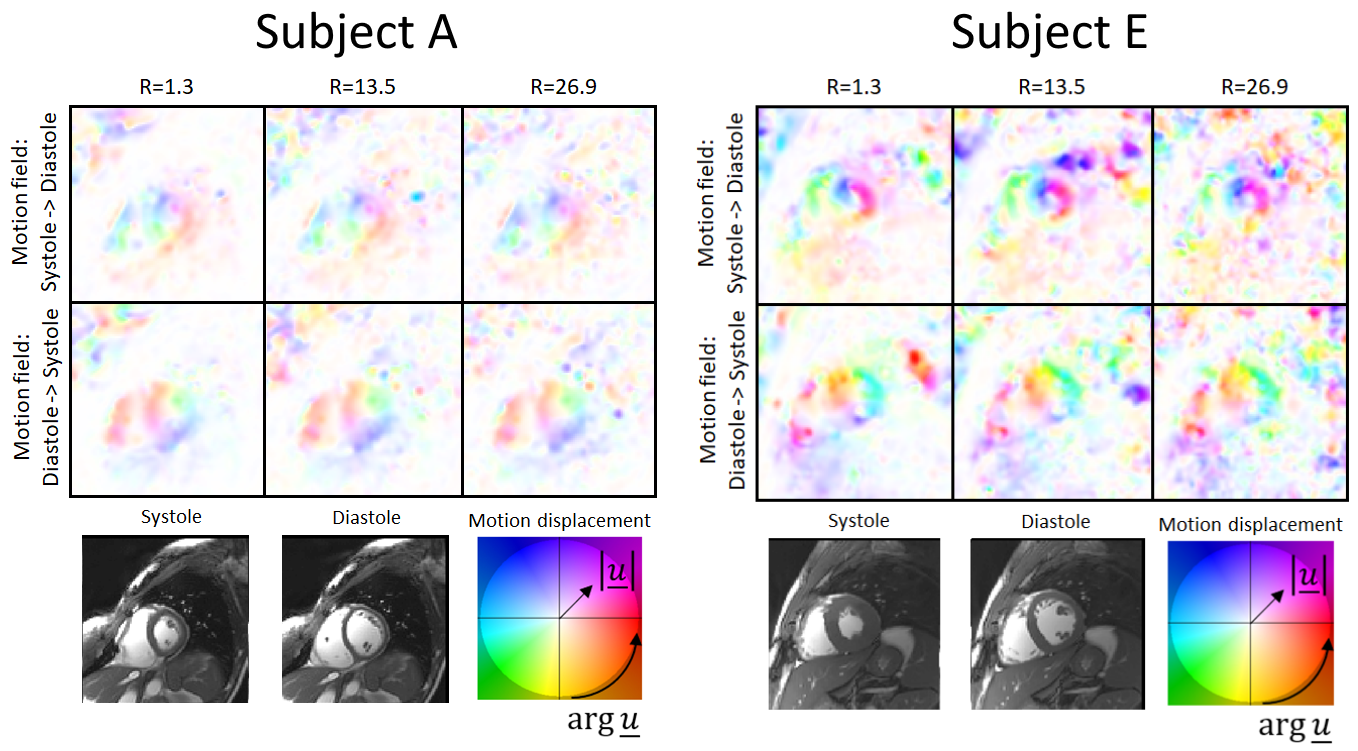

Supplement: Supplementary file 1 — Figure S1. MC‐CINE estimated motion fields between systole and diastole at three different acceleration factors, for representative subjects A and E. Contractile radial motion of the left ventricle and papillary muscles is apparent in the coloured representation of the motion. Motion of the right ventricle, liver and other surrounding organs can also be observed, in contrast to the chest wall which is almost absent of motion. [file NBM-36-e4942-s002.tif]

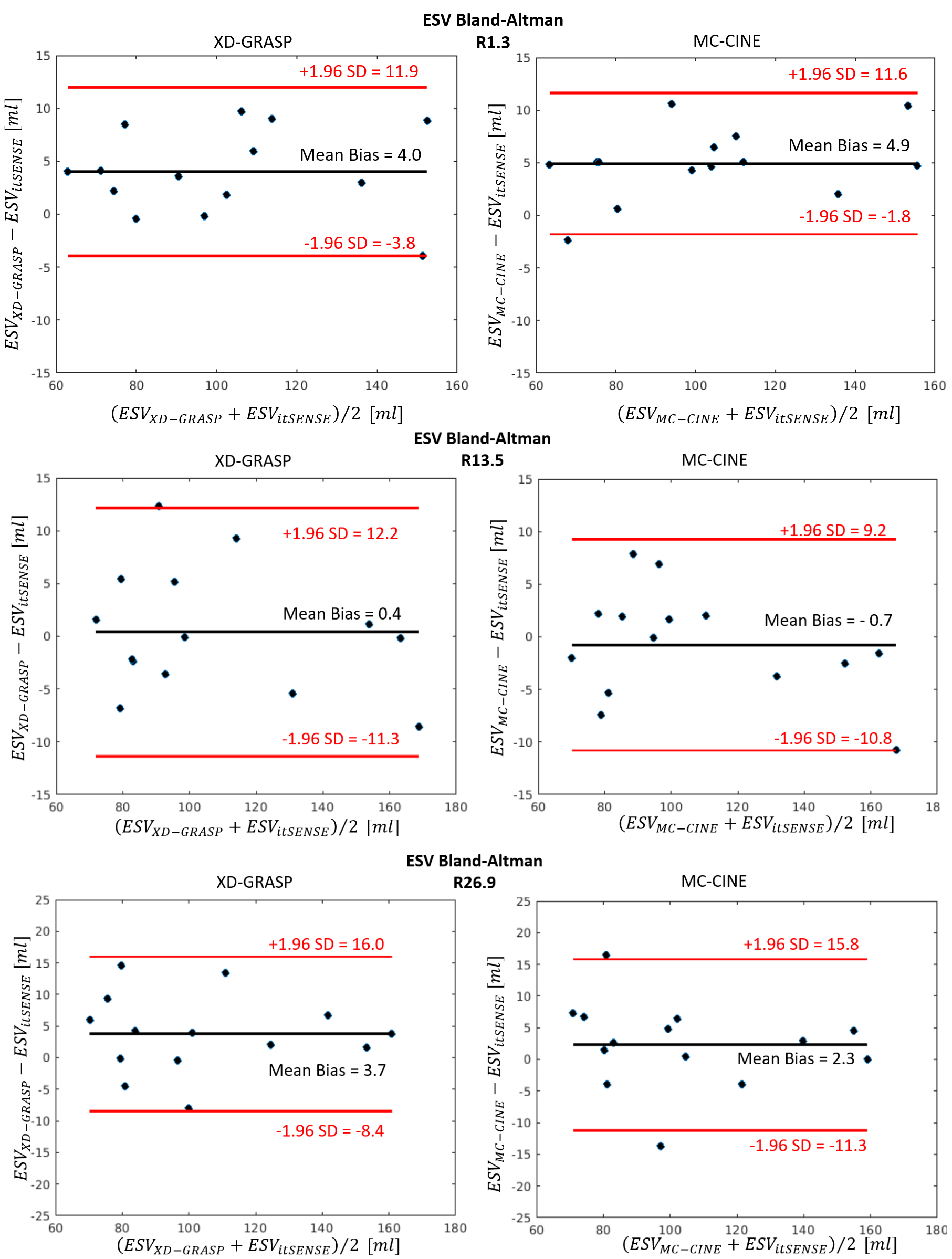

Supplement: Supplementary file 2 — Figure S2. Bland–Altman plots of the End Systolic Volume (ESV) for XD‐GRASP and MC‐CINE, relative to iterative SENSE at multiple acceleration factors. XD‐GRASP and MC‐CINE both present small and comparable biases in ESV. Similar limits of agreement were also observed for both methods across all metrics. [file NBM-36-e4942-s003.tif]

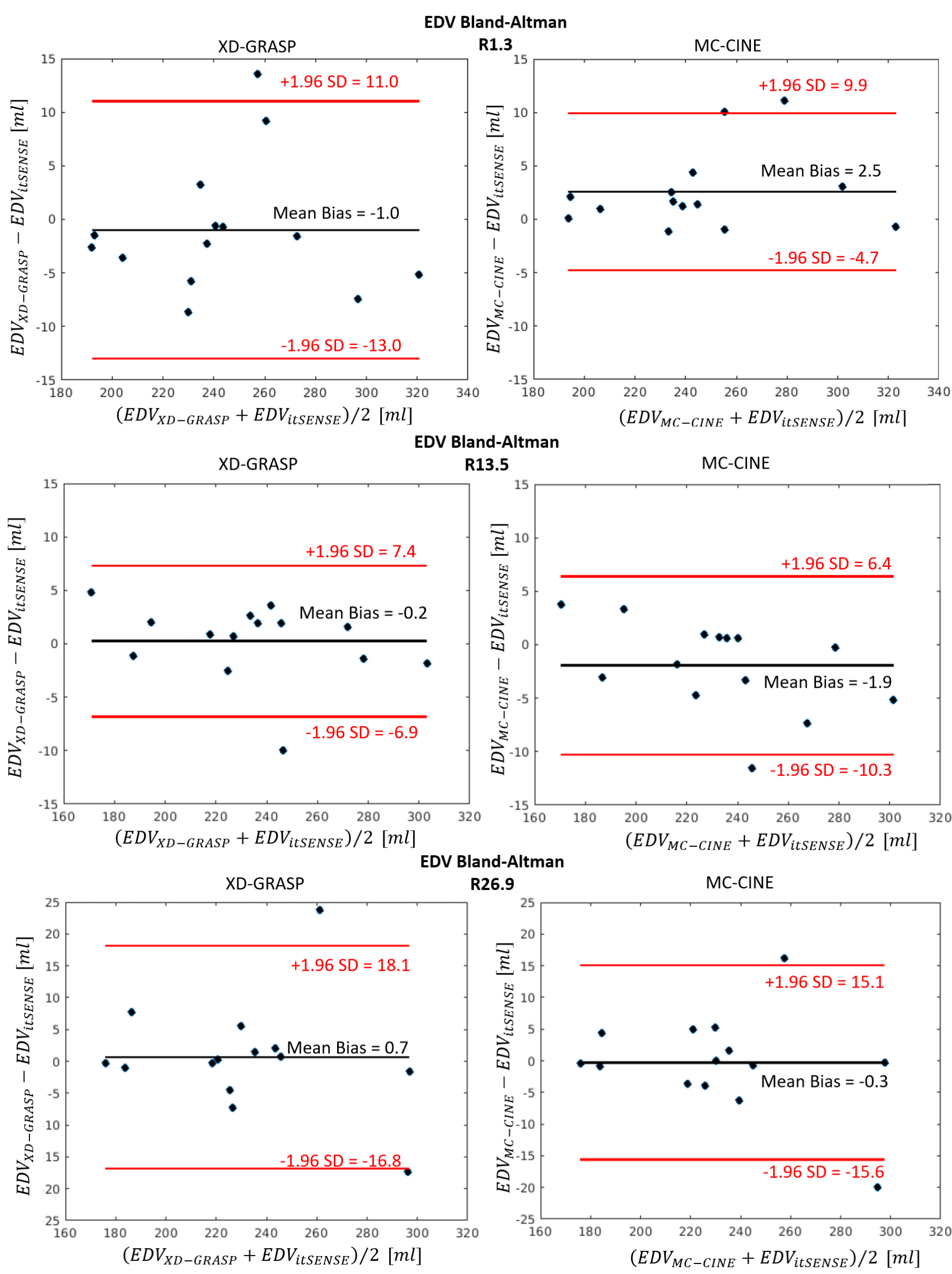

Supplement: Supplementary file 3 — Figure S3. Bland–Altman plots of the End Diastolic Volume (EDV) for XD‐GRASP and MC‐CINE, relative to iterative SENSE. XD‐GRASP and MC‐CINE both present small and comparable biases in EDV. Similar limits of agreement were also observed for both methods across all metrics. [file NBM-36-e4942-s006.tif]

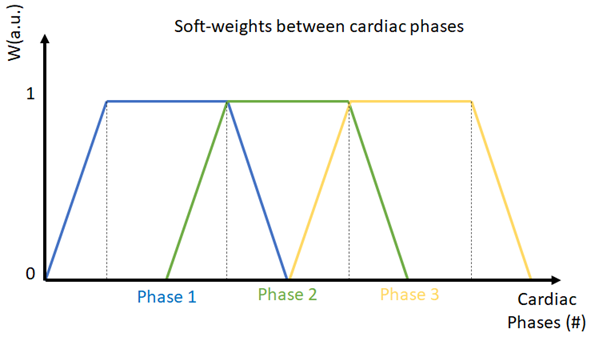

Supplement: Supplementary file 4 — Figure S4. Soft‐weight values used within the proposed approach, valued between zero and one. Data away from the centre of the phase is linearly weighted, allowing each data point to belong to multiple cardiac phases. [file NBM-36-e4942-s004.tif]
